# Women's Lived Experiences in the Use of Complementary and Alternative Medicine for Breast Cancer Management: A Phenomenological Study

**DOI:** 10.1177/08980101241277680

**Published:** 2024-09-12

**Authors:** Juliana Christina, Wendy Abigail, Anita De Bellis, Ann Harrington

**Affiliations:** Flinders University South Australia; 175508 Universitas Jenderal Achmad Yani; Flinders University; Flinders University; Flinders University

**Keywords:** breast cancer, complementary and alternative medicines, qualitative study, women < group/population, experience, Indonesia, holistic nursing, integrative medicine

## Abstract

This study aimed to explore women with breast cancer (WBC) lived experiences on the use of Complementary and Alternative Medicine (CAM) for breast cancer management. van Manen's phenomenology of practice was used as the methodology and method in this study. In-depth interviews guided by semi-structured questions were conducted with 21 WBC recruited using convenience sampling. The thematic analysis generated four main themes: Access, affordability and support for medical treatment, beliefs in CAM treatment, feeling the potential benefits of CAM, and Acknowledging the negative aspects of CAM. The outcomes from using CAM based on the lived experiences of WBC indicated that some CAM treatments could improve quality of life. However, some fraudulent CAM obtained from unprofessional CAM providers could cause harmful effects, delay medical cancer treatment, and increase breast cancer treatment costs. Therefore, there is an urgent need to enhance the awareness of appropriate treatment, including evidence-based CAM, for WBC. Improved understanding in the use of CAM as a part of quality breast cancer care services could contribute to increasing the quality of life and survival rates of women with breast cancer.

## Introduction

The use of complementary and alternative medicine (CAM) has become a global phenomenon in the general population worldwide, particularly among the group of people diagnosed with cancer (Choi et al., 2023; Tangkiatkumjai et al., 2020). As a collection of non-mainstream therapies and health approaches (NCCIH, 2021), CAMs are considered as ‘complementary therapy’ when they are used alongside standard medical treatment and as ‘alternative medicine’ to substitute or replace medical treatment (National Cancer Institute, 2022). The use of CAM is particularly prevalent among women with breast cancer (WBC) in both high-income countries and low-middle-income countries (Hammersen et al., 2020; Nayeri et al., 2020; Pandey et al., 2021; Rinihapsari et al., 2022).

In most high-income countries the use of CAM such as products to strengthen the immune system, meditation, acupuncture, yoga, massage therapy, naturopathy, and dietary supplements with vitamins and /or minerals are provided by professional practitioners (Behzadmehr et al., 2020; Hammersen et al., 2020). In contrast, WBC in low and upper-middle-income countries commonly obtain CAM from traditional healers or shamans. In this instance, CAM includes herbal remedies, massage, ayurveda, unani, siddha, supernatural power healing rituals, and spiritual therapy such as prayer and Qur’an recitation therapy (Ernawati et al., 2020; Pandey et al., 2021; Razali et al., 2020; Sobri et al., 2021; Tobing et al., 2020).

In Indonesia, for example, breast cancer is the first leading cause of cancer death in women and they were more likely to use at least one form of CAM for breast cancer management (Ferlay et al., 2024; Gautama, 2022; Tobing et al., 2020).The most favorable CAM used by Indonesian WBC were herbal therapy, traditional medicines, *kerokan* (scraping the skin of the sine), supernatural therapy and Islamic spiritual therapies due to their affordability and accessibility (Basry et al., 2022; Solikhah et al., 2020; Tobing et al., 2020). However, scientific evidence on the efficacy and safety of such CAMs to be used for primary breast cancer was limited and insufficient. Some CAM therapies, particularly herbal products, may be unsafe as they may contain toxic compounds that could interfere with medical cancer treatments (White, 2020). The use of CAM could also cause unpleasant side effects that could negatively impact the quality of life (Tangkiatkumjai et al., 2020). Therefore, this phenomenological study aimed to explore WBC's experiences using CAM for breast cancer management in Indonesia.

## Method

van Manen's phenomenology of practice was conducted to understand, describe and interpret the lived experiences of Indonesian women in the use of CAM for breast cancer management. The existential methods developed by van Manen and van Manen (2021) of lived self-other (relationality), lived body (corporeality), lived space (spatiality), lived time (temporality), and lived things (materiality) were applied. In this research, the concept of relationality reflected how participants experienced connections or relationships with others about the use of CAM. The corporeality concept was used to reflect what the participants physically felt and experienced about the use of CAM for breast cancer management. The concept of spatiality was utilised to reveal what participants experienced about space regarding the use of CAM. The concept of temporality guided the reflection of how participants experienced time in relation to the use of CAM. The materiality concept reflected particular things that participants experienced about the use of CAM for breast cancer management. These five existential themes were used as a guide to reflect the structure of the women with breast cancer themes of lifeworld on the use of CAM for breast cancer management. Finally, this study was reported in line with the COREQ (Consolidated criteria for reporting qualitative research) checklist.

### Ethics Approval

The study was approved by the Social and Behavioral and Ethics Committee (SBREC) Flinders University, South Australia, with approval number 7792. Permission letters for data collection processes were obtained from the chair of cancer support groups in Indonesia. All the information in relation to respect for the participants’ autonomy was written in the letter of introduction, information sheet and consent forms.

### Research Settings and Participants

Women with breast cancer (WBC) who had experience in the use of CAM at two cancer support groups in Indonesia were recruited using purposive and snowball sampling techniques. Thirty-four WBC were interested in participating in the study. However, four women did not meet the inclusion criteria and seven withdrew from the study due to personal reasons or poor health conditions. In total, 21 WBC were selected based on the inclusion criteria: 1) had been diagnosed with breast cancer at any stage, 2) had experiences in the use of CAM, and 3) able to communicate in the Bahasa Indonesia language. See Table 1 for the characteristics of the participants.

**Table 1.** Characteristics of Participants

ID	Age (years)	Stage of cancer	Level of education	Occupation	Type of CAMs used for breast cancer management
P1	49	III	High school	Unemployed	Acupuncture, ginger tea, ginseng potion from Shinse, and acupressure
P2	42	II	Primary school	Unemployed	Soursop leaves, papaya leaves, Binahong (heart leaves), herbal therapy
P3	41	IV	Primary school	Unemployed	Herbal therapy, traditional Chinese medicine, herbal potion from Shaman
P4	43	IV	High school	Islam spiritual teacher	Chinese herbal therapy, germanium stone therapy
P5	29	IV	High school	Canteen staff	Traditional therapy from Shaman, herbal therapy, soursop leaves, sarindam benalu kopi (macrosolen cochincinensis leaves), jelly gamat (sea cucumber) spirulina, propolis, snake head fish capsules
P6	51	IIIB	High school	Unemployed	Dog meat soup, snake meat soup, deer placenta capsules
P7	59	I	High school	Retail shop owner	Herbal therapy
P8	52	IIB	Bachelor's degree	Restaurant owner	Herbal therapy from Shaman, Chinese herbal medicine from Sinshe
P9	52	IV	High school	Cleaner	Holy water from Ustad (Islam spiritual leader), Chinese herbal therapy from Sinshe, homemade herbal concoction, germanium stone therapy
P10	59	III	Primary school	Unemployed	Daun mengkudu (Noni leaves), soursop leaves, daun mahkota dewa (phaleria macrocarpa), sarindam benalu kopi (macrosolen cochincinensis leaves)
P11	51	IV	Primary school	Unemployed	Soursop leaves, sarindam benalu kopi (macrosolen cochincinensis leaves), sambiloto (Andrographis paniculata), tumbuhan sarang semut (myrmecodia pendans), spiritual therapy from Ustad (prayed holy water, holy verses from Quran)
P12	57	III	Primary school	Unemployed	Soursop leaves, sarindam benalu kopi (macrosolen cochincinensis leaves), sambiloto (Andrographis paniculata), cassava
P13	42	II	Diploma	Unemployed	Jamu (traditional Indonesian medicine), ginger tea
P14	64	III	Diploma	Unemployed	Soursop leaves, Bawang Dayak (eleutherine bulbosa capsules), herbal remedy
P15	52	III	Bachelor's degree	Unemployed	Soursop leaves, mangosteen peel, sarindam benalu kopi (macrosolen cochincinensis leaves)
P16	65	I	Diploma degree	Book editor	Meditation, music and dance therapy, and traditional potion from Shaman contained fermented swallow bird nest, sarang semut (myrmecodia pendans), noni fruit, turmeric, snakes venom
P17	23	III	Diploma	Casual teacher	Traditional Chinese medicine, soursop leaves, tumbuhan sarang semut (myrmecodia pendans), round-rooted galangal, meditation
P18	53	IIIA	Bachelor's degree	Volunteer	Traditional Chinese medicine, herbal therapy, ecct vest (electro-capacitive cancer therapy), energy therapy, coffee enema therapy, baking soda therapy and green tea therapy
P19	38	II	Diploma degree	Unemployed	Spiritual therapy, energy therapy
P20	49	IIIA	Bachelor's degree	Wedding gallery owner	Soursop leaves, Islamic spiritual therapy, honey, propolis
P21	57	I	Bachelor's degree	Volunteer	Soursop leaves, turmeric juice, traditional Chinese medicine, massage

### Data Collection and Analysis

The principal researcher gathered the WBC lived experiences regarding the use of CAM for breast cancer management data through 30–45-min face-to-face in-depth interviews. The researcher developed a semi-structured open-ended questions schedule to guide the interviews. The interview questions ranged from general questions such as demographic data such as age, educational background, stage of breast cancer, length of time diagnosed with breast cancer and occupation to specific questions including how their experiences and knowledge in the use of CAM. The specific questions, for instance, ‘Could you tell me about your experience of using CAM?’ and ‘Could you tell me what it is like, or what it feels like to use CAM?’ The interviews were conducted in convenient places for participants, including their residence and a meeting room in the support group office. Only the principal researcher and participants attended the interviews. The principal researcher explained the details of the study and allowed participants to ask questions. The participants read and signed the consent form prior to the interviews. The interviews were conducted in Bahasa language and digitally audio recorded. The termination of interviews occurred after data saturation had been reached. The principal researcher transcribed the interviews and translated them into English, and two professional translators reviewed the accuracy of the translations. A manual thematic analysis process approach that involved isolating thematic statements and composing linguistic transformations, as suggested by van Manen and van Manen (2021), was applied by the principal researcher. Isolating thematic statements refers to uncovering thematic aspects from the lived experience descriptions using holistic, selective, and detailed reading approaches. Composing linguistic transformations is a creative hermeneutic process of writing notes and paragraphs based on reading and other research activities. This process is a part of research activities that captures the thematic statements of the identified themes in more phenomenological sensitive paragraphs (van Manen & van Manen, 2021). The four expressions of rigour, including balance integration, openness, concreteness and resonance, were used to describe the quality of the hermeneutic phenomenological text in this study (De Witt & Ploeg, 2006).

### Findings

Four main themes emerged from the data analysis of the lived experiences of WBC in Indonesia. These are, as follows:

#### Theme 1: Lack of Access, Financial Constraints and Family Support to Use Medical Treatments

The lived experiences of WBC indicated that lack of access, inability to pay for medical treatment, and family support were the main reasons for the use of CAM for breast cancer management. Nine of the WBC who lived in both remote and rural areas reported that travel distance had limited their access to cancer medical treatment, which was generally provided in metropolitan hospitals. Accordingly, CAM was commonly opted for as a primary treatment for breast cancer as highlighted by this participant:

The doctor said I must immediately seek cancer treatment in a cancer centre located in Jakarta (the capital city). I was very confused because I had to travel that far from this island…So, I first used CAM to heal my breast cancer because I had no money. I couldn’t afford travel and accommodation expenses to come to the public hospital in the city. The travel expenses from my village to get there for two years of treatment is not small. I could not get chemotherapy because of my financial situation that's why I use CAM to treat my breast cancer (P8).

Participants were more likely to use CAM for breast cancer management due to financial constraints. As reported by participants, travelling from remote and rural regions to seek medical treatment in metropolitan hospitals required additional expenses such as transportation costs, accommodation, and meals. However, the majority of the participants were unemployed due to their breast cancer condition, had no personal income, and relied on their family's support for funding their cancer treatment. Financial support from their family was also very limited and insufficient to cover cancer treatment costs and other related expenses. Therefore, the participants utilized CAM as an alternative therapy to substitute primary breast cancer treatment. One of the participants claimed:

Women seek alternative treatment for many reasons. The first reason is financial problems. The medical treatment effect was instant, but at that time, I did not have enough money to afford it. I needed to prepare money for my medical expenses and living costs during the treatment… While I was saving money for medical treatment, I had to take action to reduce the pain in my breast. Therefore, I used an alternative treatment because I did not have enough money and was not ready to undergo medical treatment. So, for me, an alternative treatment is a substitute therapy while waiting for access to medical treatment (P13).

The majority of participants expressed that their family members, including husbands, children, parents, and siblings, influenced the use of CAM. Since most participants financially relied on family support, they felt obliged to obey their family's expectations to show respect and appreciation for their support. The participants stated:

My husband stated, ‘Don’t go for surgery straight away’. He suggested praying first to request Allah to show the best treatment option. My husband disagreed with the tumour removal surgery even though I was ready for it. But I had to respect my husband's decision and I had to get his permission for the surgery. I asked my husband why he disagreed if I had surgery. Whether he was afraid of what I would become because I lost my breast? Actually, he was not afraid of it. But he would agree if the surgery was the only way treatment that I should have. My husband said, ‘We are not searching for other treatment options’. I became uncertain about having surgery due to my husband's disagreement… My husband supported me in making the decision to use alternative treatment. So, I used CAM to respect my husband's decision (P18).

My parents sent benalu kopi [loranthus] from Samosir, my village, to Medan (the capital city of North Sumatra), then from Medan to Batam Island. I didn’t pay anything for it. My mother gave money to my uncle to get benalu kopi for me from the forest in the village. Sometimes she gave IDR 20.000 ($2.00) to my uncle for a big shopping bag of benalu kopi [loranthus]…Honestly, I use herbal therapy because my mother sent it to me. I appreciate her effort to support me. I would not feel good if I didn’t use it. Also, my sister recommended I drink moral berry leaf extract. She knows someone who has recovered from cancer by taking moral berry leaf extract. So, I had drunk moral berry leaves before I drank benalu kopi [loranthus] extract (P5).

Overall, the participants were very dependent on their families for support. The WBC lacked confidence in making decisions about medical treatments without their family's consent.

#### Theme 2: Insufficient Knowledge, Fear of Medical Treatment, and Unfounded Belief

Insufficient knowledge and fear of cancer medical treatment, as well as having unfounded beliefs about CAM, were identified as other common reasons for the use of CAM for breast cancer management. Lack of knowledge was described by participants in regard to the procedure for breast tumor removal. Participants believed that invasive medical treatment such as surgery would stimulate the breast tumor to become more aggressive and grow faster. This lack of understanding led some of the participants to avoid surgery giving preference for CAM use. One of the participants stated:

Cancer cells are like trees. If we pruned a tree, the roots would grow stronger. Similarly, if we trimmed a plantation or flower, the branches and leaves would grow faster. I don’t want my cancer to grow and get bigger. I believe that when the cancer is touched by a surgical blade, the cancer cells will grow fast and spread, so I should avoid surgery and use CAM instead as CAM has no harmful effect (P20).

A lack of knowledge about breast cancer and its early detection led the WBC to ignore early breast cancer symptoms. Many participants perceived that breast abnormalities were symptoms of general disease, rather than cancer. This in turn caused delay in seeking further breast examinations. Instead of seeking medical assistance, participants treated their symptoms with CAM therapies such as massage therapy, herbal potions or by applying ointment onto their breasts. By the time they sought medical treatment, the breast cancer was at an advanced stage. One of the participants stated:

Before my lump got bigger, I had felt a light sharp pain in my breast, but I just ignored it. I thought it would go by itself. Most elderly said a breast lump is the only gas that is stuck in the breast, so I was not concerned about this. I drank an herbal potion given by a traditional healer and she massaged the lump. Then, when I was too tired of doing my daily activities, I had a fever, and the lump got bigger. Finally, I went to a hospital to check the lump. A doctor in the hospital said it was only a mammary gland inflammation, but it ended up being breast cancer (P2).

A lack of knowledge about oncological treatments also caused fear of breast tumor surgery and fear of chemotherapy for most participants in this study. As reported, they often witnessed family members, friends and other women die following breast tumor surgery and chemotherapy. These negative experiences led them to refuse or delay surgery giving preference to using CAM to shrink their breast tumor naturally, as participants claimed:

I have seen many people around me die of chemotherapy. One of my sisters had cancer. She had chemotherapy for one year then she died. Even my mother passed away three months after breast tumor surgery I also heard one of our community members passed away due to chemotherapy she had for breast cancer… It seems that all the medical treatments were not helpful and must have side effects. That makes me scared, and I am not brave enough to use medical treatment. But I have never heard of people dying because of using alternative therapy for breast cancer that's why I chose alternative therapy. I preferred using ginger therapy from a Shaman. The Shaman tapped grated ginger onto my breast to shrink the lump naturally. I used alternative therapy. I think if I have chemotherapy, I will die, and if I do not have chemotherapy, I will die as well. So, I prefer not to have chemotherapy rather than suffering from its side effects. Having chemotherapy or not, I will surely die so I am better enjoying my life without the side effects of medical treatment (P4).

The majority of participants also used CAM based on their unfounded beliefs. Unfounded beliefs referred to participants accepting rumours or recommendations encouraging them to use non-evidence-based CAM for their breast cancer. These falsities were generally received from their families and friends (who had previously had cancer), social media, or religious leaders. A strong motivation was the implication that they could get an immediate cure. This resulted in contributing to their belief that CAM was the best option and even a cure, as stated by a participant:

My brother produced soursop herbal capsules by himself. This herbal therapy has proven to be effective on his wife, who had been diagnosed with breast cancer by an oncologist…But then his wife consumed soursop herbal capsules. She believes and is sure that the herbal therapy would work. After two weeks of consuming the soursop herbal capsules, she had a medical check-up in a hospital. The medical examination result showed that the cancer had gone. Even the doctor was so surprised to see that the cancer had disappeared…This made me believe in and be confident in using soursop herbal therapy. All my friends’ experiences in using herbal therapy made me believe it…I know the evidence of the efficacy of herbal therapy from my family experiences who first used herbal therapy. If I did not see the evidence, I would not believe it. This is real, I witnessed that herbal therapy helped my family and friends (P2).

The WBC unfounded beliefs on the use of CAM were also constructed by their religion and faith. Most of the participants were Islam, and they believed that Islamic meditation ‘*dhikir*’, prayer therapy, herbs, germanium stone were natural therapies from Allah (God). These religious belief-based therapies were more trusted than medical treatment, as highlighted by this participant:

Well, my husband stated that treatment in Islam is based on spiritual belief and Sunnah^1^. He reminded me that surgery should be the final way…Allah created medicine in nature, so people need to be back to Allah and nature. So, until now, I believe that my doctor and medicine are from nature. For instance, Islamic meditation ‘dhikr’, prayer, breathing exercises, germanium stones, herbal and energy therapy are CAMs that bring healing from Allah. Maybe, even if I only believed in the mineral water that Allah gave me as my medicine, I would be cured. In fact, the therapy which is given to me is the alternative treatment. So, based on my personal opinion, my medicines are God's creations, such as CAMs that can truly save a human's life (P4).

In addition to religion, having faith in God's will also support the use of CAM. Participants emphasised that healing was in God's will, and if God allowed them to be cured from breast cancer, they would be cured no matter what medicines they used. Accordingly, some of the WBCs selected CAM for breast cancer management as mentioned by a participant:

But I surrender everything to God's hands. All in God's hands…If it's God's will, it will. So, energy therapy is truly developing faith in God… Even using medical treatment or other therapies will work if it is asked from God. So, I use energy therapy to ask for healing from God. Until now, I have had no obstacles to using energy therapy. I believe this treatment is from God. I support alternative therapy and other non-medical treatments as long as I use them with the belief that only God heals. I must strongly believe that God is the only one who can heal (P19).

Overall, delays in seeking medical treatment due to fear of cancer medical treatment, believed in non-evidence-based CAM would allow breast cancer to progress and worsen the WBC health condition.

#### Theme 3: Experiencing the Benefit of CAM

Some participants in this study described positive outcomes and potential benefits they experienced from the use of CAM. These benefits included alleviating pain, increasing physical and immune system strength, experiencing fewer side effects, and that the cost was more affordable than medical treatment. Further they believed that CAM could be used for cancer prevention.

According to participants, CAM was identified as an effective analgesic in reducing breast cancer pain. As explained by one participant, the use of soursop herbal capsules, Jamu (herbal potion made from curcumin and herbal extracts), white turmeric, ginger therapy, mixtures of herbal potions (e.g., soursop leaves and ant-plant extract), meditation, touch therapy, and prayer significantly relieved their pain. For instance, one of the participants stated:

I experienced positive effects from consuming soursop herbal capsules. It can relieve my pain. Before taking it, I had very bad pain throughout all my body…But after taking soursop herbal capsules the pain is not the worst. Also, my breast pain decreased by approximately 50% after drinking Jamu (an Indonesian herbal potion made from curcumin and herbal extract). Similar to Jamu, ginger therapy could reduce my breast pain as well. Ginger is even more effective than others. The breast lump got smaller. Yes, there were some changes I felt from using it. I felt a hot sensation when the grated ginger was applied to my breast. The breast was very painful; the pain was relieved by the heat from the ginger (P2).

Overall, the participants’ personal experiences indicated that CAM potentially had natural analgesic properties. However, the participants did not mention the reduction of pain levels or the duration of the pain relief effect. Other potential benefits of CAM mentioned by the participants were increased physical strength and boosting their immune systems.

Some of the participants experienced fatigue, tiredness and a weakened immune system after receiving chemotherapy. In order to reduce the side effects of chemotherapy, the WBC consumed CAM, such as herbal extracts (which they obtained from traditional healers*),* honey and animal-based food supplements. For example, one of the participants revealed that she consumed dog and snake meat soup or deer placenta capsules to maintain her immunity, strength and energy during her chemotherapy. This woman claimed that these CAM treatments increased her physical endurance as demonstrated in the following excerpt:

I was recommended to drink dog meat soup. I think you have known that people in Medan eat dog meat…Yes, dog meat soup. I drank it. My leukocyte level has never fallen down since I have been consuming it. Indeed, I felt more energy after drinking dog and snake meat soup. I felt my body gets warm…Well, that helped my leukocyte at the normal level …In fact, after taking deer's placenta capsules, I felt stronger. I felt that I had more energy to manage my health condition. I still had nausea but not the worst (P6).

Overall, it is evident from the WBC experiences that CAM has the potential benefit to increase physical strength and boost the immune system during breast cancer treatment.

#### Theme 4: Acknowledging the Negative Aspects of CAM

Some participants acknowledged the adverse effects of CAM treatment. They experienced potential toxicity, felt the treatment was futile, and doubted its safety. The potential toxicity of CAM experienced by participants included a hot sensation in their throat whilst consuming soursop leaf extract, and when CAM providers applied herbal therapy ointment onto the site of the breast tumor, burning pain. The following excerpt indicates how a participant experienced the side effects of the CAM treatments:

…But when I drank it [soursop leaves] frequently, I got a sore throat like an irritation. I had to stop drinking it because I couldn’t normally swallow after drinking the Soursop extract…Then he [the therapist] applied a grated cassava onto my breast to reduce the heat. Sometimes, I felt a hot and stabbing pain like being punctured with many needles. I felt a hot sensation in my body after taking the internal alternative radiotherapy that contained snake venom. The therapist explained that the hot sensation was from the snake venom (P12).

More severe adverse events that participants experienced after consuming herbal therapy included nausea, vomiting, dizziness, burning pain in the stomach, constipation, and kidney and liver disease. Examples of these expressed symptoms are as follows:

The side effects of herbal therapy that I experienced were feeling sick, like vomiting, and nausea. Also, I could not normally defecate after consuming Sarindam Kopi (Loranthus paraciticus). The stool was solid. My stomach was distended. But I think it's from my poor health and it's my body's reaction, so I keep drinking it because I want to recover (P3).

…After 20 days of having the Chinese herbal [therapy] as a substitute for chemotherapy, my whole body becomes swollen and full of fluid. I did blood tests, and the results showed that I had a liver and kidney problem. I stopped the herbal therapy immediately…I thought it was normal to experience side effects of herbal medicine (P20).

Other participants realized the futility of CAM once their breast cancer had not healed and had metastasized into other areas of the body. The delay in the use of medical treatment due to the use of CAM in the initial instance resulted in the breast cancer developing to advanced stages, which may have been otherwise successfully treated using oncological treatments. The participants also claimed the use of CAM had wasted their money and their time, but this realization did not come initially, only after trial and error. One of the participants stated:

After six months of consuming the herbal capsules, I checked my CA (cancer antigen) 3 level and found it increased to 1.4 U/mL. Later after eight months, the CA level rose to 6.4 U/mL…it [the cancer cells] was increasing instead of decreasing. So, I thought I should not play with this. If the CA level increased up to 6.4 U/mL, the cancer could be metastasised…Obviously, the herbal therapy was not helping at all. According to doctors, cancer cells cannot be killed by herbal therapy. It might have a potential substance that can be used to treat cancer, but the reaction process is very slow. However, cancer cells develop very fast. This statement made me realise that herbal therapy could not cure cancer (P7).

I drank the herbal potion from each Sinshe^2^ for up to one month. When I found that there were no significant changes, I stopped and changed to herbal therapy from others Sinshe. In six months, I had sought treatment from all Sinshes in Batam…It was only wasting money and time. When the cancer stage got to the advanced stage or stage 4, the cure would be more difficult…I did not feel any reactions from using the herbal capsules even though its so many, approximately six different types of capsules. I took every one of those five to seven herbal capsules a day…There was no effect at all. I just felt very dissatisfied (P8).

Legality and safety concerns regarding CAM were also mentioned by the participants after realizing the treatment they used was ineffective. Three of the participants emphasised that the government should control CAM treatment practices and registration in Indonesia, since they had become aware of many questionable CAM practices provided in this country. One participant stated that some CAM therapists created a fraudulent CAM training certificate in order to register their practice and obtain a permit to provide CAM practices:

So far, I know several alternative therapists who professed themselves as medical doctors. I don’t know whether this is true or not. They might have a fake certificate, which is created using a Photoshop editor application. It is easy. But in fact, what I see is no new changes in the development of traditional treatment. The government has acknowledged this but is not concerned about this… The government must control alternative treatment CAM providers’ permits. For instance, when I was given plant roots to treat cancer, the government should know whether the safety of the plant roots has been scientifically tested or not. I think this procedure has not been included in the process of getting the alternative treatment registration. This can be a disadvantage for people with cancer. Therefore, the government should not legalise a doubtful alternative treatment provider. The procedures and medicines that are used in alternative treatment should be investigated and reviewed before the permit to run the treatment is given. It is important to offer evidence-based alternative treatment (P17).

Based on the participants’ experiences, they viewed that the use of questionable CAM practices harmed their health and the questionable CAM practices financially exploited them by providing futile CAM treatments. One participant mentioned two names of the most well-known CAM therapists in their local area who provided cancer treatment illegally. This participant claimed the CAM clinics owned by these therapists were closed by the local health department due to violating the CAM regulations of Indonesia. Despite this action, these two well-known CAM providers continued to practice CAM therapies claiming publicly that they could cure cancer. The CAM providers gained profits from the CAM practices, but the participants felt they did not gain any health benefits:

Due to the sharing of deceitful information, an alternative therapy practice was closed by the local health department. However, I heard that the therapist of that practice still runs his practice illegally by moving from one place to another place. Another example of an illegal alternative practice that I knew is provided by [name of the traditional healer therapist]. There were many women with breast cancer who used her counterfeit alternative cancer therapy. They paid about IDR 3.000.000 ($ 300) for the alternative treatment. This traditional healer was very famous. She has many alternative medicine clinics nearly everywhere. To gain more money from the traditional treatment practice, she paid for a TV program to publish her treatment. Unfortunately, when she was on the TV program, she gave a presentation using a patient's medical test results. Then, professional medical doctors complained to her because she was not a medical doctor who had the competency to do medical assessments. Then the local health department closed her clinics (P21).

Participants’ experiences above indicated that some CAM therapists exploited them by giving them a sense of false hope and encouraging them to use ineffective cancer treatments. The CAM practices were profitable to the CAM providers but disadvantaged the participants.

## Discussion

The majority of participants first sought and used CAM with the aim of curing breast cancer, and seeking medical treatment was commonly considered as a second option after realizing that CAM could not permanently cure. Clearly, CAM was commonly used as a primary treatment for breast cancer instead of as a complementary therapy. However, the use of CAM can be integrated with medical treatment as it has been regulated by the Ministry of Health of Indonesia (Kementrian Kesehatan Republik Indonesia, 2018). Therefore, WBC awareness toward the use of medical CAM as a complementary instead of primary treatment for breast cancer should be increased.

Deep description and interpretation of participants’ experiences demonstrate that lack of access to medical treatment, poverty, knowledge, religious beliefs, and cultural practices contributed to the use of CAM for breast cancer management. Due to poverty, participants in this study, particularly those who lived in remote and rural areas, used CAM since this non-medical treatment was perceived to be more accessible and less expensive compared to medical treatment. Previous studies also found that the use of CAM was highly required by WBC with poor economic status and limited financial resources in lower-middle countries including Indonesia (Basry et al., 2022; Huraerah, 2019; Hutajulu et al., 2022; Rahayu et al., 2020; Razali et al., 2020; Rinihapsari et al., 2022). Personal income affects the utilization of medical treatment in Indonesia (Hutajulu et al., 2022). In addition, the use of traditional medicine in Indonesia is also expected due to the inequality of healthcare resources distribution in Indonesia and the limited access to the healthcare system (Basry et al., 2022; Rahayu et al., 2020; Solikhah et al., 2020). Therefore, access to quality healthcare should be increased and expanded for WBC as every individual has the right to equitable healthcare services (Levit et al., 2020). Financial assistance such as a monetary allowance for travel costs, medical funding resources, and affordable health insurance should be made available for poor WBC, particularly those who live in remote and rural regions. These options would allow them to access appropriate proven medical cancer treatment and relieve financial burdens (Sobri et al., 2021; Susilowati & Afiyanti, 2021). The government or private finance organizations could offer medical treatment grants or medical loans to assist WBC in paying for oncological treatment costs.

Fear of surgery and chemotherapy due to insufficient knowledge about medical treatment delayed the participants from seeking substantive medical breast cancer treatments. Previous studies showed that the use of CAM delayed medical cancer treatment, resulting in adverse outcomes including increased cancer stage development and declining health conditions (Solikhah et al., 2020). Therefore, breast health education programs and accurate information from healthcare professionals are essential to increase WBC understanding of oncological treatment (Alsharif, 2021; Sari & Rukmi, 2021). A greater understanding of breast cancer and its treatment could alleviate fears and increase confidence in the use of medical treatment to cure breast cancer. Psycho-oncological support may also be provided for WBC to overcome anxiety and fear of oncological treatments.

Most participants were from the Islamic faith and used spiritual-based CAM to obey their husbands’ decisions and family preferences. Other studies also explained the vital role of husbands in making decisions as obligated in Islamic law and patriarchal traditions, which most Islamic women, including these participants in Indonesia have adopted. Their views had a significant impact on the use of CAM (Widiasih & Nelson, 2021). In addition, having a belief and faith in God's will led participants to use CAM and impeded them from seeking medical treatment. Hence, breast cancer education should also be provided to the husband, other family members and spiritual leaders. Their support for the use of both medical cancer treatment and CAM as adjunctive therapy could contribute to increased survival rates and quality of life for women with breast cancer.

Positive outcomes from using CAM, as reported by participants, were to relieve breast cancer symptoms, reduce side effects of chemotherapy and prevent cancer cell relapse. The consumption of soursop leaves was expressed as a reduction in pain and breast inflammation, shrinking breast lumps and dry purulent breast tumor and accelerating breast wound healing. Participants believed consuming snake and dog meat soup increased physical strength and improved immune systems during chemotherapy. Taking snake meat soup and snake venom-based remedies could shrink their breast tumor and kill cancer cells. They believed reading the Quran, and praying evoked relaxation, relieved stress and sickness and strengthened their faith, promoting a positive outcome from the healing process. Several studies showed the use of CAM to be beneficial and safe and hence could improve women with breast cancer's general well-being (Behzadmehr et al., 2020; Ernawati et al., 2020; Hammersen et al., 2020; Komariah et al., 2020; Muhammad et al., 2022; Nayeri et al., 2020; Solehati et al., 2020; Zulkarnain et al., 2021). Based on these experiences, participants perceived that relieving symptoms could cure them from breast cancer. In fact, authors report that some CAM therapies might provide temporary therapeutic effects to relieve breast cancer symptoms but they would not successfully treat breast cancer (Basry et al., 2022; Nayeri et al., 2020). The use of CAM to reduce recurrence risk was an unrealistic expectation as long-term use of CAM without medical treatment potentially increases the risk of cancer developing to advanced stages and causing severe health conditions (Basry et al., 2022; Sobri et al., 2021; Solikhah et al., 2020).

Participants who did not experience any significant positive outcomes from using CAM revealed the futility of CAM. This finding is in line with studies that found there was no significant correlation between the use of CAM and quality of life (Fjær et al., 2020; Mokhtari-Hessari & Montazeri, 2020). Additionally, based on the WBC experiences, the use of CAM for an extended period increased their cancer antigen levels where the breast cancer spread into other organs such as the liver, colon, and bones, resulting in these cancers being incurable. The WBC also experienced adverse events such as nausea, stomach burning pain, and soreness after consuming soursop leaf extract (soursop contains neurotoxic acetogenin compounds)(Smith & Shejwalkar, 2020). Overall, the use of CAM potentially has adverse effects that may lead to disease progression, increased risk of death and decreased survival rates for women with breast cancer (Lin et al., 2020; Okaiyeto & Oguntibeju, 2021).Therefore, further evidence from rigorous clinical experiments on humans, such as large random controlled trials, is required to assess the effectiveness and outcomes of these treatment methods.

Participants generally obtained CAM from providers who had no license or permit to provide CAM practice legally. As reported by participants, the unprofessional CAM providers commonly advertised and promoted fraudulent CAM products as alternative treatments to cure cancer via social media, magazines, television, radio and the internet. They delivered deceptive information such as stating that CAM was the cheapest and safest medicine to cure cancer naturally. Similarly, a previous study in the US also found that misinformation on cancer treatment spread faster through social (Lazard et al., 2023). The WBCs in Indonesia, who had low education and knowledge levels, were often the common victims of receiving misleading information about the use of CAM, which led to inappropriate CAM cancer treatments and physical and financial disadvantage. Misleading information can cause potential harm that negatively affects cancer patients including women with breast cancer (Johnson et al., 2021)Therefore, surveillance of CAM practices should be enhanced to ensure that WBC is not involved in malpractice events. Increasing the availability of professionally trained and registered CAM providers would help WBC receive best-practice evidence-based CAM therapies.

Complementary and Alternative Medicine can improve the quality of life for WBC; however, it is important for WBC to be aware and acknowledge that CAM will not cure their breast cancer. Accordingly, some CAM that are known to be safe should be offered as an adjunctive therapy to improve quality of life rather than alternative medicine to cure breast cancer. It is essential for WBC to be aware of the outcomes of care from using CAM, as this could help them in making decisions regarding the most appropriate breast cancer treatment and the most appropriate CAM that could be used as an adjunctive therapy. More studies are required to investigate specific outcomes from the use of CAM as an alternative medicine for breast cancer management.

## Limitations of the Study

The first limitation of this study was the small sample sizes in each of the two provinces. This small sample size would not be adequate to reflect the entirety of women with breast cancer experiences in Indonesia, as each province in this country has a variety of similar and differing cultural contexts and religious beliefs. Unverified interview transcriptions were the second limitation. The researcher offered each participant an opportunity to review the interview transcripts and check their credibility and accuracy. However, none of the participants were willing to read their interview transcripts due to personal reasons. The third limitation was the interview transcripts translation process between Bahasa and English, which might have impacted the data interpretation. Even though great care was taken and the translations were officially checked, interpretations could have altered the original use or structure of the translated words in this cross-language study. Therefore, a loss of meaning could potentially have occurred in the translation process and affected the interpretation.

## Implications for Holistic Nursing Practice

The findings of this study highlight that there is a high demand for CAM services for breast cancer management. This indicates that there are great prospective career opportunities for nurses, such as CAM practitioners, holistic nursing providers, CAM educator roles, and researchers in Indonesia. This study recommends that nurses in holistic nursing care, particularly in Indonesia, need to enhance their knowledge and skills in the fields of CAM so that they can obtain the required license and Certificate of Competencies to be eligible to provide scientifically based CAM. More formal accredited CAM education programs are required to train nurses with appropriate knowledge and skills. Therefore, this study recommends that a CAM curriculum should be introduced to nursing students and that they be trained to identify high-quality CAM recommendations from reliable information sources. Nursing students at advanced levels, such as at the master's degree and doctorate levels, should be encouraged to conduct scientific research in CAM fields.

In addition, nurses have a vital role in providing care, support, and recommendations on the use of CAM for patients with cancer (Kusunoki et al., 2023). To provide the best quality patient care, nurses should encourage WBC to disclose the use of CAM as some CAM therapies may interact with medical cancer treatment and may cause harmful effects as experienced by participants in this study. Information, recommendations, and support given by nurses may assist WBC in making decisions regarding the appropriate use of CAM. The use of CAM should be assessed and well documented by nurses as this assessment could inform potential CAM therapy use that could be included in nursing care plans.

It is also recommended that an official evidence-based CAM information website be established in Indonesia. This website may be funded and maintained by the Indonesian government or reputable national or international organizations that specifically focus on CAM. This website could be used by nurses and other health professionals to provide current evidence-based CAM information and align with international CAM providers, as well as be a reliable information source for those who are interested in using and administering CAM. The use of evidence-based CAM provided by professional CAM providers could positively impact the health outcomes for WBC.

## Implications for Holistic Nursing Science Globally

There is an urgent need to increase access, knowledge, and awareness about the importance of appropriate medical cancer treatment, as these would contribute to reducing the number of WBC dying prematurely due to inappropriate treatments. It is evident from the WBC lived experiences in this study that CAM as a primary treatment was ineffective in curing breast cancer. Nevertheless, some CAM has potential therapeutic effects that may benefit WBC and improve their quality of life while undergoing medical breast cancer treatment. As such, suitably safe CAM should be used as an adjunctive therapy instead of an alternative medicine to treat breast cancer. The potential benefits of CAM experienced by participants indicated that CAM could possibly be used as a pre-medical cancer treatment for those who cannot have direct access to medical cancer treatment. Therefore, further rigorous scientific research related to CAM use in human studies is required to investigate the safety, efficacy and beneficial outcomes of CAM, particularly for breast cancer management. Ultimately, the findings from this study also provide invaluable insight that can be used as a source of information for holistic nurses with different cultural backgrounds to lead CAM knowledge and practice development across various healthcare settings globally.

## Conclusion

The use of van Manen's hermeneutic phenomenology approach in this study allowed the explorations of WBC lived experiences in using CAM for breast cancer management. It was evident that CAM should not be used as a primary therapy to cure/treat breast cancer. Instead, CAM can be considered as an adjunctive therapy or as an option for palliative care, particularly for WBC who are at the terminal stage of the disease, have untreatable breast cancer or refuse medical cancer treatment. The use of CAM as an adjunctive therapy to manage the side effects of chemotherapy should be supervised by healthcare professionals including holistic care nurses. The recommendations offered from this study could contribute to better outcomes related to breast cancer treatment, including CAM, which WBC used, and hence, increase survival rates and improve the quality of life of women with breast cancer.

## Notes

1. Sunnah is Islamic religion doctrine.

2. Sinshe is a Chinese traditional medicine healer.
